# Supplementary material for: Post-lockdown abatement of COVID-19 by fast periodic switching
Source: PLoS Comput Biol. 2021 Jan 21;17(1):e1008604. doi: 10.1371/journal.pcbi.1008604 (PMC7861565; doi:10.1371/journal.pcbi.1008604)
Supplement: Attachment — Submitted filename: Replies_Reviewers.pdf [file pcbi.1008604.s002.pdf]

Replies to the Reviewers' Comments

Title: Post-Lockdown Abatement of COVID-19 by Fast Periodic Switching

Authors: M. Bin, P. Cheung, E. Crisostomi, P. Ferraro, H. Lhachemi, R. Murray-Smith, C. Myant, T. Parisini, R. Shorten, S. Stein, L. Stone

Submitted for possible publication to

PLOS Computational Biology

Paper no. PCOMPBIOL-D-20-01341

Comments of Deputy and Associate Editors

Comment: As with all papers reviewed by the journal, your manuscript was reviewed by members of the editorial board and by several independent reviewers. In light of the reviews (below this email), we would like to invite the resubmission of a revised version that takes into account the reviewers' comments. In particular, we would like to see attention to the questions of parameter sensitivity, relative levels of activity, possibility of resonance, and clarification of the results and discussion. On the conceptual front, referee 2 points out that the main idea and associated results are somewhat "obvious". Can the authors better articulate the ways in which the mathematical/computational analysis contributes in a non-obvious way?

Reply: We would like to thank the Editors for providing insight on the main issues in our original manuscript. We also thank the Reviewers and Editors for their time in reading and evaluating our manuscript. The reply document is organised as follows.

- Each of points raised by Reviewers are replied to individually.
- Generally, numbered indices referring to specific publications in the Reply document indicate those listed in the bibliographical references from the original manuscript. We also include new references in the present document. These are explicitly mentioned in the text.
- For the Reviewers' convenience, each individual point raised in the Reviewers' comments is listed. As per the instructions in the decision letter and again for the Reviewers' convenience we also submit the revised paper with the changes highlighted in blue font.
- We have added extra material to explain the comment regarding the obvious nature of the idea. First, it is not obvious (from the theory of switched systems) that average dynamics will be approximated with periods of the order of days. This only happens under very fast switching conditions. That *very fast* corresponds to periods of the order of one week, is truly an interesting finding. New theoretical results are included to underpin this, as well as other mathematical findings. Finally, an outer feedback loop is presented, and theoretical results given to underpin this design.

Comments of Reviewer 1

Comment 1.1: Summary comments: No model source code or data was submitted for download as a zip file, Github link or in any other format. As a result we are unable to determine the reproducibility of the work presented.

Reply 1.1: The Reviewer is right and we apologise for not having provided the SW code at time of original submission. All code is now available on Github at https://github.com/V4p1d/FPSP_Covid19.

Comments of Reviewer 2

Comment 2.1: My first and most serious concern is that the paper proposes a switching strategy to exit from a coronavirus induced lockdown. The conclusion - that the net effect is the weighted average of the dynamics during and out of lockdown is reasonable. Although, I would've expected some level of resonance with the incubation period of the disease.

Reply 2.1: Consideration of the issue of synchronisation/resonance is important and we have now added material in the revised manuscript to make the reader aware of how this issue is addressed by our scheme (subsection "Unmodelled synchronisation effects" on p. 26). We repeat some of this discussion here.

In principle, there are several pathways in which resonance effects may possibly manifest themselves, all associated with uncertainties (although as we explain, we don't believe they play a major role in the context of our work). Before discussing some of these we make the following specific comments.

(a) As we have mentioned, our system includes an outer feedback loop which is specifically designed to mitigate possible instabilities or undesirable behaviour that may emerge due to delays and uncertainty. It is important to note that the outer-loop not only serves to cautiously adapt the duty-cycle based on the observed data filtered over a given suitably long time-window as shown in Fig. 5, but also effectively acts as a safety valve which will close should levels of average rate of infections exceed a specified threshold (see the new Fig. 9 and 10 in the revised Sensitivity analysis section on p. 24).

(b) We also note that we have tested our strategy on many models, stochastic, deterministic, agent-based, for both SIQR and SEIQR based models. For a wide variety of models, with realistic distributions of incubation time and infectivity profile, simulations show that the effects of synchronisation and resonance give rise to either very small effects or are non-existent.

With regard to the latter point, we note that the issue of resonance in SIR based models has in fact been discussed in several papers. For example, our simulations would appear to be consistent with:

N. Bacaër, X. Abdurahman, Resonance of the epidemic threshold in a periodic environment, J. Math. Biol., 57: 649-673, 2008.

which discusses the non-existence of resonance in certain SEIR and SIR models with exponentially distributed recovery times near the bifurcation point where $\mathcal{R}_0 = 1$. In contrast to the above paper, other recent analytical investigations focus on SIR models which, when forced with large amplitude oscillations at a multiple frequency of the model's intrinsic frequency, are capable of generating parametric resonance. For instance, see

S. Chen, B. Epureanu, Regular biennial cycles in epidemics caused by parametric resonance, Journal of Theoretical Biology, 415: 137-144, 2017.

In our context, this can occur when the period of the forcing or lockdowns (here, of the order of weeks) is an exact multiple of the period of the intrinsic dynamics of the system (of the order of 6 months). Thus, because of the frequency mismatch, we can't expect the lockdowns to entrain the far longer time-scale of the natural epidemic dynamics.

As the Reviewer points out (Reviewer 3 made a similar observation), one might suspect the possibility of resonance or synchrony with the incubation period. In fact, the intrinsic period/frequency of SIR models are only partially governed by the incubation period, and are in fact greatly controlled by the infectives recovery period (as studies of the intrinsic frequencies of these models show), and the level of mixing. It is possible that delays due to incubation introduce some natural frequencies in the unforced model. However, realistic distributions of the incubation and infectivity time are spread over days (from several days to nearly two weeks). This, in turn, results in the fact that the modes associated with such frequencies are very damped, and do not lead to any appreciable synchronisation effect.

For complete transparency, we note that one could construct artificial situations in which synchronisation is perhaps an issue. For example, were the incubation period to be an exact, fixed time-delay (which it definitely is not), one might theorize that this may act to synchronize the incubation period with the FPSP open period i.e., after lockdown. In such cases, our outer-loop has been designed to remove this synchronisation and increase the open period to abate the virus. It is also worth noting that, even without the outer loop, this effect would also shunt all symptomatic infectives into the lockdown period where they could be quarantined. Such a synchrony could act to eradicate the disease in a possibly efficient manner. However, such scenarios are very unlikely as the incubation and infectivity periods are not fixed delays, but rather given by reasonably broad time distributions. Furthermore, household infections during lockdowns, which are considerable, will further act to abate any synchrony of this form.

Finally, even though we do not expect significant synchronization effects to occur in practice, resonances are highly complex phenomena and appear when least expected, often by complete surprise. In the unlikely case that synchronisation between the disease dynamics and the FPSP policy were to emerge due to the complexity of the disease dynamics, human behaviour, or the many time-delayed processes and dependencies that might trigger synchronization, the slow measurement-based outer-loop which controls and regulates the long-term dynamics of the FPSP procedure. The "outer loop" prevents any unmodelled resonance effect by changing the lockdown period in such a way as to suppress any large infection peaks that manifest themselves reducing activity back to full lockdown if required.

To illustrate the *safety-valve* nature of the outer-loop, we conclude Reply 2.1 by presenting an example that we did not include in the original manuscript. In this example we judiciously choose incubation times in an SEIR scenario to give rise to a rapidly growing infection. Specifically we select the incubation to be Gamma-distributed (this code is available in the manuscript code repository [1]) with a narrow variance. Note that these distributions effectively approximate a pulse and are actually completely unrealistic - i.e. chosen with very low recovery variance. Note that such distributions are also not captured by our simulation models. However, as can be observed, despite this adversarial choice of parameters, the slow outer supervisory control suppresses the growth in the I state (Fig. 1, bottom) by just slightly decreasing the switching duty-cycle.

[1]https://github.com/V4p1d/FPSP_Covid19/

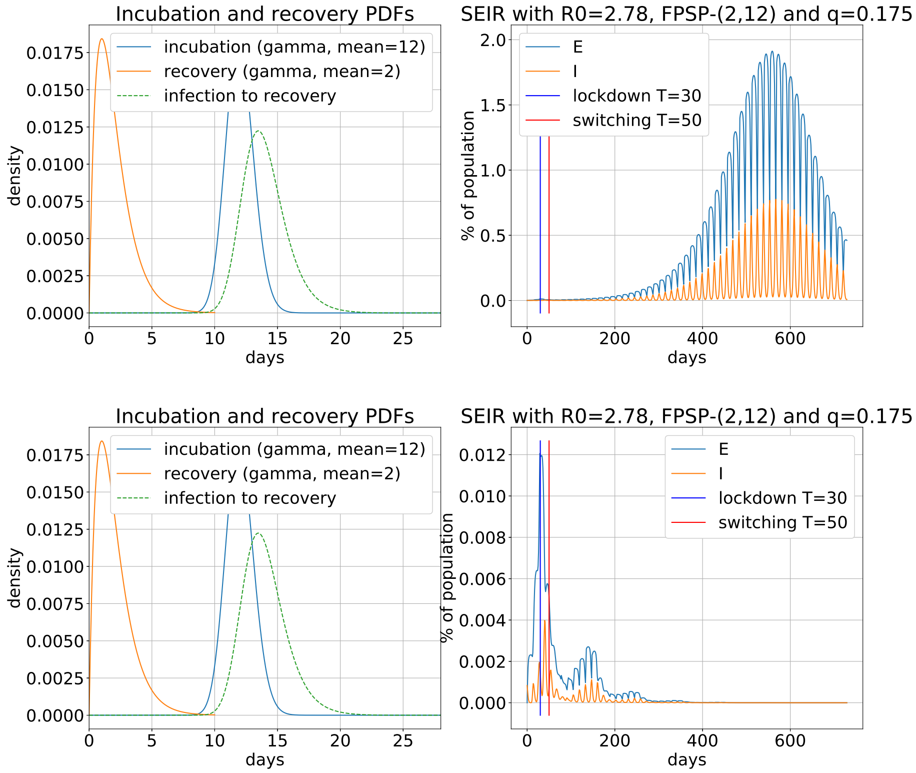

Figure 1: Fast periodic switching with open-loop policy (top) and with slow outer supervisory control (bottom) applied to an SEIR model with Gamma-distributed incubation and recovery period. Plots in the left column show distributions of incubation time, recovery time, and total time between infection and recovery used in simulation. Plots in the right column show the percentage of exposed and infectious individuals over time. Despite low variance incubation and low variance recovery after incubation, the slow outer supervisory control adjusts the duty-cycle to suppress growth of the I state.

Comment 2.2: Although, I would've expected some level of resonance with the incubation period of the disease. However, my issue is that this could easily be misinterpreted by a willing public. That is, the model assume lockdown of n days followed by free movement for m days to allow for partial economic reactivation - although no economic modelling is done. My concern is that the authors assume that the populace will be only as active in the m days of free movement than they would be on a random day without lockdown (and therefore disease spread and economic recovery can both be fractional). A more natural (to me) assumption is that the population will overcompensate during limited periods of "freedom" and exacerbate infection further than predicted here.

Reply 2.2: Increased activity during "open" periods was in fact discussed as part of the sensitivity analyses in the original manuscript. We apologise if this was not clear and we hope it is now. In particular, we draw the Reviewer's attention to Fig 9 of the original manuscript, which was an explicit analysis of the overcompensation possibility. We perform sensitivity analysis of the concurrently infected population (the quantity $I + D + A + R + T$ in the SIDARTHE model) on increased infection rate $(1 + d)\beta^+$ during periodic active days, where d is the level of increased

activity. Again, we apologise if this was not clear in the original manuscript and we have hopefully made this clearer in the revised manuscript. In this respect, we emphasise that, compared with the original manuscript, in the revised paper the sensitivity analysis results have been revised and expanded in that Figs 9 and 10 now also include the outer-loop effects (see p. 24-26 and p. 37 in the Appendix of the revised paper).

Notwithstanding the above comment, the point made by the Reviewer is extremely important. In fact, in our opinion, the most concerning of all the synchronisation effects may be interactions between the FPSP policy and the behaviour of the population. In this setting, behaviour may drive adaption of the FPSP, and adaption of the FPSP may drive a change in behaviour. In principle, this is a classical resonance pathway where individual parts of the system excite and *pump* each other. There are however, several aspects of the FPSP policy that may serve to dampen the impact of this interaction. First, regular periodic open intervals, with short lockdown intervals between these open intervals, may mitigate interactions due to reduced urgency with which individuals utilise open intervals. Second, during open intervals, we expect that the following holds: (i) the presence of additional measures, such as the usage of masks and social distancing policies preventing the overcompensation suggested by the Reviewer; (ii) a high level of compliance in the population with general health policies to keep the level of mixing to a manageable level. Such levels of compliance, may of course, be enforced by policy and law. Moreover, if people compensate by scheduling all their chores to take place during the non-lockdown days, thus increasing the value of β^+, then this also implies that they are less active during lockdown days, thus decreasing the value of β^-. In fact we have used values of β^- estimated during a period of full lockdown when, in the absence of open intervals, some basic level of activities were always permitted). Since it is the average of the two values that counts, this in theory implies there will be no effect. Finally, again we note the outer feedback-loop. Interaction of the outer loop with the population is designed in a manner to drive the population towards a full lockdown if negative effects manifest themselves due to increased mixing. Knowledge of this fact may induce or encourage good behaviour in the population; if it does not, the unstable interaction between FPSP and the population will drive the system to an equilibrium that suppresses the virus anyway (i.e., the full lockdown state). Namely, the outer-loop guarantees a safety by enforcing, in the worst case, a full lockdown (see again above Fig. 1 in the present document and the new Figs 9 and 10 in the revised section on Sensitivity Analysis on p. 24).

Comment 2.3: The principle contribution of this work is a mathematical analysis of an extended SIR model (SIDARTHE) with application to a switching parameter space and the (rather obvious) consequence that the switching between two sets of parameter values effectively acts as a duty cycle. The mathematical analysis is fine, my comments and suggestions related primarily to the simulation and interpretation.

Reply 2.3: We would like to thank the Reviewer for their comment. We have added some comments to the introduction to explain the non-obvious aspects of the FPSP strategy. First, switching may actually induce unstable or even chaotic behaviour and average behaviour of the *dynamics* only follows if one switches fast enough. That periods of one week correspond to very fast switching is a very useful finding for this class of models. Second, the theoretical results are new and not obvious. Moreover, a further truly non-obvious component of our work is the inclusion of a regulating outer loop and the theoretical results underpinning this. Please note again that the fast-switching control law coupled with the slow measurement-based outer-loop may induce different forms of transient behaviour and these are not obvious a-priori, differing significantly from other recent low-frequency

approaches such as, for instance, [9] in the original manuscript. The averaging effects are related to the asymptotic behaviour as per our theoretical results, and these also constitute an important guideline for a designer.

Comment 2.4: My second principle concern is that there is no sensitivity analysis of the presumed parameters of the model.

Reply 2.4: Actually, the original manuscript indeed reported a sensitivity analysis section on page 21 in the context of the Materials and Method section. We apologise if this was not clear. In particular, unfortunately, Fig. 10 was missing above the caption due to a technical glitch in producing the final manuscript, and we apologise for this oversight. In any case, we further extend our sensitivity analysis in the revised manuscript (see Figures 9-10 and associated discussion reported in sections "Sensitivity analysis" and "Policy options with FPSP" on p. 24-28). More specifically, we analyse the behaviour of the open-loop FPSP and of the supervisory outer control loop under varying estimates of β^- (see Figs 9, 11 (centre), and 12 (centre)), β^+ (see Figs 10, 11 (right), and 12 (right)), $\mathcal{R}_0$ (see Figs 11 (left) and 12 (left)), switching period (see Fig 12), duty-cycle (see Fig 11), and uncertainty in the joint distribution over β^+, β^-, σ_1, ... σ_{16} (see Fig 14 in the Appendix).

Comment 2.5: comparison to season effects is spurious as the frequency is much lower (line 42)

Reply 2.5: We would like to thank the Reviewer for this comment. The comment is indeed correct. However, please note that our motivation in making these comments is to assign proper credit to those who have gone before us. While it is true that our switching strategy is a much higher frequency than other seasonal studies, there are strong structural similarities. We have revised this section of the paper to make our motivation clearer.

Comment 2.6: There other typos throughout, a careful proofreading would help.

Reply 2.6: We have proof-read and corrected the paper. We hope it is better now.

Comment 2.7: since the author point out other similar studies [22-24] to lend authority to their model, it would be useful to more thoroughly consider what the particular unique contribution of this work is. (95-97)

Reply 2.7: We thank again the Reviewer for this comment. The unique contribution of our work is four-fold. First, to the best of our knowledge, we are the first group to propose this strategy, with our work appearing online on arXiv on March 22nd, 2020 (reference [22] in the original manuscript, now [23] in the revised paper). Second, we give solid and thorough theoretical results to justify and inform the design of our strategy. Third, an outer loop design is proposed that provides a measurement-based cautious adaptation mechanism that deals with uncertainties and delays. This is based on rigorous control theoretical concepts. To make this more transparent, in the revised paper, we added a new subsection "(iv) Overview of outer feedback loop properties" on p. 11. Finally, the study provides a rigorous exploration of the proposed strategy, and presents tools that can be used by policy makers to better inform decisions.

Comment 2.8: - reference to *official statistics* (line 123-4) is confusing. Please say exactly what it is

within these statistics that is interesting.

Reply 2.8: We agree with the Reviewer. We revised the sentence mentioning these official governmental documents highlighting the specific facts that are relevant in our exposition.

Comment 2.9: rather than n-days on and m-days out of lockdown, why not lockdown a fraction of the population? Wouldn't this achieve the same result?

Reply 2.9: The Reviewer is again correct, and in principle, this is also an option. However, the distinct advantages of our strategy are the following. First, with our strategy, leakage between compartments is easier to manage. Epidemiological dynamics under compartmental population models, with some compartments under lockdown while others are not, are governed by cross-infection rates and therefore depend strongly on the particular choice of population compartmentalisation. Second, compliance with the strategy is easier to enforce in using a temporal strategy, rather than in a spatial one (as we are now witnessing throughout the world). We believe that a population-wide simultaneous short periodic lockdown is simpler to implement, with respect to public communication, public acceptance, and enforcement, than a permanent lockdown rule based on personal characteristics. Indeed, we emphasise that leakages are critical (i.e., small values of leakages make separation pointless) and hence going for solutions that allow a stricter separation seems preferable.

Comment 2.10:- line 281-2 Eqn (6) : there is a t missing. And, is c distinct from T? - Following line, you're assuming that a

Reply 2.10: We thank for the first comment where indeed the Reviewer is correct, and c should have been T. This has been corrected.

Comment 2.11: - line 309-310: why do you need H and E as separate categories? It serves no purpose here and just complicates the model.

Reply 2.11: We understand this comment by the Reviewer. Actually, this is a feature of the specific model we use for the validation of our mitigation strategy. The SIDARTHE model (reference [17] in the original manuscript, now [18] in the revised paper) is used in our context as a benchmark to explore the effectiveness of our strategy. We used the model *as is*; proposing improvements would be beyond the scope of the present work. However, for complete transparency, the new section "Unmodelled synchronisation effects" on p. 26 of the revised paper discusses some of the elements that are missing in the SIDARTHE and other related COVID-19 models, and their effects.

Comment 2.12: - line 327-333: what effect does these parameter choices have on your results? Where do they come from and do they matter?

Reply 2.12: The effect of the parameter choice on the epidemic and their impact on our results are explored in "Sensitivity analysis" on p. 24-26 and 37-38 (see. Figs. 9-12, 14 and the associated discussion). The particular set of parameters used in the main body of the manuscript was taken from [17] (now [18] in the revised paper) as we emphasize in the main text. These parameters correspond to those fitted to data observed early in the COVID-19 pandemic in the Lombardy region in Italy.

<u>Comments of Reviewer 3</u>

Comment 3.1 : Given that the optimal pulsating period for the lock-down is around a week, and the incubation period of the virus is between 1 and 2 weeks, Have you considered the possibility of a resonance between the two oscillations (lockdown and incidence) which could make matters worse? After all, this synchronous release of lock down will force all new infections to happen within a narrow time window creating delayed wave of incidence. This does not show up on your results, but couldn't it be because the SIDARTHE model does not include a E (exposed) compartment? If it did, perhaps the ratio between of the incubation and lockdown periods could become an important parameter to consider.

In addition, Reviewer 3 also adds:

this synchronous release of lock down will force all new infections to happen within a narrow time window creating delayed wave of incidence. This does not show up on your results, but couldn't it be because the SIDARTHE model does not include a E (exposed) compartment? If it did, perhaps the ratio between of the incubation and lockdown periods could become an important parameter to consider.

Reply 3.1: Please see above discussion on resonance (Reviewer 2), in which we would like to highlight Fig. 1, and the additional section in our manuscript (subsection "Unmodelled synchronisation effects" on p. 26). In addition to these comments, the omission of the E-class in our simulation validation results and its justification should be better explained.

Our principal validation tool is the SIDARTHE model which was calibrated using measurements from Lombardy in early Spring, 2020. SIDARTHE corresponds to a strict generalisation of the SIQR model. The disease dynamic is very challenging in the SIQR setting. Here, a susceptible is immediately exposed to infectives, and the disease grows rapidly. This scenario thus provides a robust environment for testing the FPSP policy and the outer feedback loop. However, it should also be noted that the performance of our FPSP policy is not overly sensitive to the specific assumptions of the adopted validation model, and may be applied in other model settings. For instance, we have also tested our policies in SEIQR environments, using stochastic differential equations, as well as agent based simulations, in which the exponential rates are replaced by more realistic distributions, and in which the E class plays a role in the disease dynamics.

We also note that there are several other important unmodelled effects that could, in principle, lead to the same or similar effects, mentioned by the Reviewer. For example, the assumption of exponential departures and arrivals between classes is, of course, a gross approximation (as we mentioned in line 707 of the original manuscript). In addition to incorporating the outer-loop to mitigate this uncertainty based on actual measurements, a large part of our existing sensitivity analyses is concerned with quantifying the impact of more realistic distributions in our validation models. This part of the paper has now been expanded, and the results discussed in much more detail (see sections "Sensitivity analysis" and "Policy options with FPSP" on p. 24-26 and also on p. 37-38). It is worth noting that none of these give rise to significant resonance effects in any of our simulations based on realistic modelling scenarios and assumptions.

Finally, we also note again that the outer-loop is designed to increase the length of lockdown, relative to open-days, to suppress any increase in infection levels that may arise due to the afore-

mentioned or other unmodelled effects. The design of this feedback loop is such that any unstable interaction between FPSP disease dynamics will drive the system to an equilibrium that suppresses the virus anyway (i.e., if needed, all the way to a the full lockdown state) as shown in the new Figs 9 and 10. Namely, the outer loop guarantees a safety by enforcing, in the worst case, a full lockdown. As a final comment in reply to the comment of Reviewer 3, it is worth also noting that the authors of the SIDARTHE model justify the omission of a *strict* E-class by citing *the mounting evidence that an infected individual can transmit the virus at an early, pre-clinical stage of the disease ..., and the recent studies estimated median serial interval values for COVID-19 to be close to or shorter than the [short] median incubation period, further proving the possibility of pre-symptomatic transmission of the disease.* Even though this may (or may not) be true, the inclusion of the outer-loop effectively insures that our strategy is robust to this assumption.

Comment 3.2: Given that the optimal pulsating period for the lock-down is around a week, and the incubation period of the virus is between 1 and 2 weeks, Have you considered the possibility of a resonance between the two oscillations (lockdown and incidence) which could make matters worse? After all, this synchronous release of lock down will force all new infections to happen within a narrow time window creating delayed wave of incidence. This does not show up on your results, but couldn't it be because the SIDARTHE model does not include a E (exposed) compartment? If it did, perhaps the ratio between of the incubation and lockdown periods could become an important parameter to consider.

Reply 3.2: Please see above comments on Resonance. We make only one specific comment here; the strategy will not force all new infections to occur during open periods; rather infections will still occur during lockdowns, at a reduced rate. This is important as lockdown periods in our strategy correspond to reduce rates of mixing.

Comment 3:3: Does working intermittently actual solves the economic problem? I guess the benefits will vary depending on the type of economic activity. For example: for one of the most affected sectors, Tourism, opening for 2 days every week is not sustainable.

Reply 3.3: We have added some comments in the Discussion and in the Results sections to reflect that for some sectors it may not be feasible to remain closed for any period at all (for example, schools). That being said, the FPSP strategy can still be applied to the remaining sectors of society. Related to this, we note also that an important choice concerns the number of compartments that society can be broken up into (giving rise to a compartmentalised switching model). Please note that we have analysed such scenarios, but these remain outside of the scope of the present paper (the objective of which is to present this basic ideas underpinning our work). Please note also we have now included a section "Policy options with FPSP" on p. 25-26 (see Figs. 11, 12 and the related discussion) and contour plots to indicate to policy makers the suite of available choices when designing an intervention. These choices may of course be based on the social and economic utility of a specific intervention. Finally, we note again that the basic idea under-pinning our work is to devise a strategy to drive the epidemic to zero in a systematic manner, which is also predictable, so that social and economic activity may take place in a reasonably planned manner. A full economic quantification of the benefits of our strategy is however beyond the scope of the present paper. As a final comment we have developed a S/W tool to explore the range of situations possible using FPSP and this is referred to in the paper.

Comment 3.4: Supplementary material is missing, even thought it is referred to in line 668 Figure

10 is missing. without it it is not possible to check the sensitivity of the model to uncertainty in the parameters.

Reply 3.4: Actually, the original manuscript indeed reported an extensive sensitivity analysis section on page 21 in the context of the "Materials and Methods" section. There is no "Supplementary Information" section in this paper. Unfortunately, Fig. 10 was missing above the caption due to a technical glitch in producing the final manuscript. We apologise for this oversight. In any case, we further extend our sensitivity analysis in the revised manuscript see sections "Sensitivity analysis" and "Policy options with FPSP" on p. 24-28 and p. 37 in the Appendix with particular reference to Figures 9-12, 14 and the associated discussion).

Comment 3.5: Periodicity is also related to patterns of approaching endemicity, not just disease die-out.

Reply 3.6: The Reviewer is correct. The change of seasons has been shown to induce recurrent epidemic dynamics, that are often annual in pattern. We have now revised the relevant paragraph in the Introduction.

Comments of Reviewer 4

Comment 4.1 : It would be valuable for the authors to provide further intuitive explanation for these results, as well as their explicit connection with other systems. For example, when I read the article the first thing that came to mind was the results of periodic forced systems in physics (signals, circuits, etc). It is known that the amplitude of an oscillation will decrease when the forced frequencies are faster than its natural frequency, and that it increases as the forcing frequency gets closer to the natural one. (Does the result on the best X and Y combination depend on N?)

Reply 4.1: We expanded the Results and the Discussion sections to better characterise our contributions. The objective of this work is to design a mitigation strategy that is composed of a fast-switching mechanism that is predictable and is not directly measurement-driven, so that economic and social activity can take place in a predictable manner. We complement this basic strategy with a slow and cautious duty-cycle adaptation mechanism that indeed depends on measurements (filtering them over a reasonably long time-window). This allows us to achieve adaptation to the possibly changing situations. In this regard, we also substantially revised the Sensitivity Analysis in the "Methods and Material" Section. We hope the Reviewer finds this revised version easier to follow.

As far as the comment on oscillations is concerned, our mechanism of control is to use fast-switching to approximate an average behaviour. It is well known, from the theory of switched systems, that switching may induce oscillations due to switching, or an averaging behaviour, corresponding to very fast switching. It is this latter behaviour that is exploited in this paper. One of the principal and surprising results in the paper is to show that switching on the time scales of days/weeks gives rise to this very fast behaviour; theoretical and validation results are provided to substantiate this statement. We have now added an extra reference and comment to link this work to that of switched systems as the work is most related to this branch of engineering/applied mathematics.

Concerning Reviewer's question *Does the result on the best X and Y combination depend on N*, we would like to mention that the effect of a given choice of X and Y on the epidemics, for the class of models considered in this paper, evolution does not depend on N. In fact, the same policy can be applied to a "rescaled" model where the value of the states $S(t), I(t), \ldots$ coincide with the percentage of individuals in each compartment without any change in the time-evolution. We make reference to a number N of individuals only for the sake of exposition, but our validation remains valid if percentages are used instead of absolute numbers of individuals.

Comment 4.2 : Although the basic idea is nice and clean, it took me a while to understand the paper. The reading of the text could be cleared in some places. I provide specific examples for improving the clarity below.

Introduction

- This section is clear but is a bit long and repetitive.
- In some parts, the authors make somewhat strong statements which should be treated with care. See here examples:
- Line 58: high fidelity model? what do you mean? It might be better to avoid terms that can have

different interpretations. - Although the lack of uncertainties in the proposed method is stated in several parts of the text, this is not clear for me.

Your results, for example the optimal set of T, X and Y, come from a model whose parametrization is based on clinical datasets (that as you mention have many sources of errors) (moreover, model itself and model parametrization remains a challenge in this area).

Reply 4.2: We made an effort to streamline the Introduction and to remove statements to avoid possible wrong interpretations. Concerning the fourth comment, it is worth pointing out that the FPSP open-loop control law does not depend on any modelling assumption and is designed in terms of (i) a choice of the (high) frequency which is not dependent on any parameter to be estimated and (ii) a duty-cycle that is automatically adjusted by the outer feedback loop based on actual measurements. With regard to comment (i), it is important to note that any practical implementation would start as an exit from a full lockdown, using the outer-loop to find the appropriate duty cycle (and period). Thus, no model is needed in the envisaged implementation of the FPSP strategy. We apologise if this was not clear. Of course, very accurate models, should they be available, could be used to seed the outer-loop to refine its operation. However, it is true that the validation in simulation depends on a choice of a model and its parameters. In this regard, we substantially revised the Sensitivity Analysis in the "Methods and Material" Section and we also improved and expanded the "Discussion" Section.

Comment 4.3: Results - This section is a bit messy and in some places it is difficult to pull out the main message. A better organized version would improve understanding of this section. It may be helpful to add subsections with clear subtitles.

Reply 4.3: We have now broken up the Results section into separate subsections. We made an effort to improve the organisation of the paper also in the other sections. We hope that the revised manuscript is now easier to read.

Comment 4.4 - In addition, it would also be beneficial to explicitly guide the reader through the figures. Figure 5 is a good example of this (only a phrase in the text).

- Figures - font and legend should be bigger

Reply 4.4: In the revised paper we improved the presentation and discussions of figures.

Comment 4.5: Parameters need units (eq 7). For example, beta is a rate (this is specified in line 224 for example), so what does $beta = 1$ mean? if the units are days-1, that would mean that an individual only interacts with one person per day, which is hard to believe. Are $g1, g2, g3$ and $g4$ rates (line 311) or probabilities? ($g := \sigma$). If $beta$ is a rate, then $gi, i = 1, 2, 3, 4$, cannot have units and therefore cannot be a rate. Maybe I am missing something here.

Reply 4.5: The Reviewer is correct, and we thank them for pointing these inconsistencies out. The parameters σ_i are rates, with dimension 1/day. The functions β_i (or β in the SIDARTHE equation) have instead dimensionless values ranging in the interval $[\beta_i^-, \beta_i^+]$. These functions perform a scaling of the multiplied terms without altering their dimension, and they are used to model

the effect of a lockdown on the infection rates. In the whole revised manuscript, with the exception of the sensitivity analysis section, β_i^+ is taken equal 1, and $\beta_i^- < \beta_i^+$. In this way, $\beta_i(t) = \beta_i^+$ implies that the infection rates are at the 100% of their nominal value, while $\beta_i(t) = \beta_i^-$ models the effect of the lockdown. In the sensitivity analysis section, β_i^+ is taken larger than 1, to model possible effects of overcompensation. We specified this in the revised version.

Comment 4.6: I understood that these parameters are fitted from clinical data, right? Does this data set correspond to periods with intervention? If so, the model and its parameters represent a situation under intervention. Although the main goal of the paper would be independent of this, your results on the optimal T and the values of beta+ and beta- would not be right? Please, discuss or clarify this.

Reply 4.6: The Reviewer is correct. Optimal tuning of the parameters had been performed in [17] (of the original paper), based on data observed early in the COVID-19 pandemic in the Lombardy region in Italy. In particular, β^+ had been estimated in a pre-lockdown situation (when first containment rules had been advised, such as social distancing or frequently washing hands), and β^- in a lockdown situation. We agree with the Reviewer that the actual values of β, should our strategy be applied, may be different from those of a fully lockdown, or fully open, situation. We did try to account for this in our sensitivity analysis. As the Reviewer points out, we also agree that the main goal of the paper is independent of this, and, starting from a possibly conservative duty-cycle, the objective of the outer feedback loop is to adjust the duty-cycle to reflect the true evolution of the pandemic. Finally, as per the period T, we mainly consider multiples of one week (and do not allow the outer feedback loop to change it) to improve the simplicity and the convenience of applying the proposed policy.

Comment 4.7: Figure 3 is difficult to understand, please consider other options for this.

Reply 4.7: We understand the comment of the reviewer. Figure 3 is indeed difficult to digest. However, it contains a compact albeit comprehensive summary of the large number of simulation results discussed in the paper. We have now attempted to link Figure 3 (now Figure 4) with Figures 3 (the new one) and 5 to better explain the information contained therein. We hope this is more comprehensible now. If the Reviewer still feels this should be removed, we will certainly gladly remove the figures, should the paper be accepted.

Comment 4.8: Discussion is unclear.

Reply 4.8 We have restructured the Discussion on p. 28 of the revised manuscript. The main findings of the study are enunciated in a dedicated section. An expanded discussion is then given. Finally, concluding remarks are presented to highlight implications of our work, and future directions.

Comment 4.9: When you mention the FPSP for policy making, I would like to see a discussion with other options. For example, your simulations are based on a reduction of almost 85% of the contact rate which is not a small number (moreover, the used beta+ in itself could be quite small; I do not know because I do not know the units) and, in the best of cases for 5 days a week. This corresponds to an R0* of about 0.9. For this scenario, I would like to know your thoughts on the FPSP approach vs a constant "partial-reduced" beta, which for this example, would represent a reduction of 60%. So, is it

better to implement 5 days with a reduction of 85% or 7 days with a reduction of 60%?

Reply 4.9: We agree with the Reviewer on the need for providing policy makers with tools to explore competing options for policy interventions. In the revised manuscript we further extend our sensitivity analysis in the revised manuscript (see Figures 9-10 and associated discussion reported in sections "Sensitivity analysis" and "Policy options with FPSP" on p. 24-28). More specifically, we analyse the behaviour of the open-loop FPSP and of the supervisory outer control loop under varying estimates of β^- (see Figs 9, 11 (centre), and 12 (centre)), β^+ (see Figs 10, 11 (right), and 12 (right)), $\mathcal{R}_0$ (see Figs 11 (left) and 12 (left)), switching period (see Fig 12), duty-cycle (see Fig 11), and uncertainty in the joint distribution over β^+, β^-, σ_1, ... σ_{16} (see Fig 14 in the Appendix). We mention that we provide level sets in Figs. 11-12, which explicitly quantify the trade-offs between varying duty cycle and switching period lengths with respect to the peak infected population under varying infection parameters. We have also developed a software tool to assist policy makers. This is available open-source and a reference is given in the paper.

The question on whether it is better to implement a switching policy with an average reduction in infectious contacts by 60% or a policy with a constant reduction of such contacts by 60% is rather complicated, as the rate of infectious contacts can only be influenced indirectly. One avenue of active research is exploring compartmental population models, with some part of the populace under lockdown while others are not. These models are governed by cross-infection rates and therefore depend strongly on the particular choice of population compartmentalisation. We speculate that a population-wide simultaneous short periodic lockdown is simpler to implement, with respect to public communication, public acceptance, and enforcement, than a permanent lockdown rule based on personal characteristics. However, we agree that this is impractical for some economic sectors. So, decisions could be made for the case for certain critical economic sectors to be added to the 'essential' category, at the cost of an increased level of β.

Comment 4.10: Here the uncertainties appear again. Please be clear on what you mean by uncertainties.

Reply 4.10: The sources of uncertainties are now extensively discussed in section "Elaborated discussion" on p. 28 of the revised manuscript.

Comment 4.11: Empirical results? I did not see them.

Reply 4.11: In the revised manuscript, we clarified that we are referring to simulation results and we distinguish from instances of the term "empirical" when it is has the correct meaning as done, for example, in the Introduction. Moreover, we emphasise that the Results section has been revised and additional discussions and explanations have now been provided. We thank the Reviewer for pointing out the need for additional explanations.

Comment 4.12: There are parts where references are missing and sentences are vague. For example, the phrase that goes from line 638 to 641, ref? Which early results? If you mention this, you do need to explain more clearly what is different now. Another example: Line 703: "... are enough to approximate ..." what is enough? Did you define a criteria? I am curious also if the one week is robust under different population sizes and betas.

Reply 4.12: We made our best effort in revising the manuscript to add missing references where needed and to avoid vague statements. We hope it is better now.
